# New approaches for developing biomarkers of hormonal contraceptive use

**DOI:** 10.1038/s41598-022-24215-4

**Published:** 2023-01-05

**Authors:** Rakhee Sachdeva, Narender Kumar, Vivian Brache, Barbara A. Friedland, Marlena Plagianos, Shimin Zhang, Larisa Kizima, Leila Cochon, Ana Sofía Tejada Tabar, Ann Blanc, Ruth B. Merkatz

**Affiliations:** 1 Center for Biomedical Research, Population Council, 1230 York Avenue, New York, NY 10065 USA; 2 Clinica de Profamilia, Nicolas de Ovando Esq. Calle 16, Ens. Luperon, Santo Domingo, Dominican Republic; 3 Gold Canyon, USA

**Keywords:** Biological techniques, Medical research

## Abstract

To identify biomarkers of hormonal contraceptive (HC) use in urine and saliva, we conducted a pilot study with 30 women initiating levonorgestrel (LNG) containing combined oral contraceptives (COCs) or depot medroxyprogesterone acetate (DMPA) (15/group). Based on established COC pharmacokinetics, we collected serum and urine samples before COC ingestion and during Days one and three of use, or before DMPA injection and on Days 21 and 60 post-injection. We used liquid chromatography-tandem mass spectrometry (LC–MS/MS) to measure serum/urine LNG and MPA. LNG was undetectable at baseline (specificity 100%); post ingestion, most urine samples had detectable LNG levels (sensitivity: 80% 6 h post Dose one, 93% 6 h post Dose three). We used a DetectX LNG immunoassay kit and showed 100% sensitivity measuring urine LNG. Urine MPA levels were undetectable in 14/15 women at baseline (specificity 91%); post-injection all urine samples had detectable MPA levels (sensitivity: 100% days 21 and 60). Results suggest urine sampling can be used to identify a biomarker of LNG and MPA use. Based on evidence from other steroidal hormonal studies showing changes affecting the transcriptome profile of saliva at 24 h, we used the same (COC, DMPA) timepoints to collect saliva. We performed transcriptome analysis and detected several differentially expressed genes in DMPA users’ saliva on Days 21 and 60 compared to baseline; none among COC users. We plan further research of differential gene expression in saliva as a HC biomarker of DMPA use, and will explore longer periods of COC use and saliva collection times, and application of microRNA sequencing to support using saliva as a COC biomarker.

## Introduction

Accurate data about contraceptive use has implications for establishing public health goals, delivering clinical care, and conducting clinical research. In the field of public health, statistics on contraceptive prevalence rates (CPR) affect planning to achieve realistic national and international objectives for sexual and reproductive health services^1–3^. Accuracy of the CPR data is especially important in regions where pregnancy rates remain high despite reports of high rates of contraceptive use. As a result, provision of obstetrical and neonatal clinical care services may not be commensurate with local, regional, or country needs^4–6^. Access to information about contraceptive use can also facilitate shared decision-making between clinicians and clients regarding selection of a contraceptive method best suited to achieve individual family planning goals^7^. Regarding clinical trials, data obtained from volunteers about their use of a method constitute the basis for analyzing and reporting safety, efficacy, and acceptability results and are fundamental to regulatory reviews and decisions about approving new methods^8^.

Given the overall importance of contraceptive use information, numerous investigators have reflected on ongoing challenges associated with obtaining accurate data that is based on self reporting^7–13^. Recently, several investigators have described more encouraging results in obtaining accurate data but recommend use of objective measures to validate self -reported contraceptive use^14,15^. Measuring serum concentration levels of synthetic progestins (the active component of hormonal contraceptives) in samples is considered the “gold standard”^16^ for assessing hormonal contraceptive (HC) use. However, such testing requires invasive venipunctures, relies on laboratories with specific capacity to evaluate exogenous hormonal concentrations, and is impractical for use in household surveys or late-stage clinical trials with thousands of participants. Investigational approaches for measuring hormone levels in urine are being developed, although these tend to rely on sampling endogenous progesterone and estrogen (such as estradiol) in the context of clinical care^17^. As has been well described, both endogenous and synthetic steroid hormones are mainly metabolized in the liver and are partially excreted as conjugated metabolites and intact hormones in urine. We anticipated that we could use well-described and highly sensitive methods to measure these hormones or their metabolites in urine samples as markers of hormonal use^17–20^. Specifically, we speculated that we could use validated liquid chromatography-mass spectrometry (LC–MS/MS) methods to measure LNG and MPA levels in both blood and urine samples as well as the highly sensitive LNG Enzyme Immunoassay kit (DetectX; Arbor Assays, MI, USA) to measure immunoreactive LNG in urine. Hence, we sought to determine if urine, which can be collected more easily and cost-effectively than serum, could be used as an objective biomarker of commonly used contraceptives formulated with progestins, such as levonorgestrel (LNG), found in many combined oral contraceptives (COCs), or medroxyprogesterone acetate (MPA) used in injectable formulations, e.g., depot-medroxyprogesterone acetate (DMPA).

We also considered the potential of using saliva to identify biomarker/s as this body fluid is not merely a plasma ultrafiltrate but also contains an entire library of proteins, hormones, antibodies, and other molecular compounds. It is an ideal matrix for the application of transcriptomics to analyze differential gene expression^21^. We theorized that synthetic steroid hormones such as LNG and MPA enter the saliva through passive diffusion. However, their expected concentrations are low compared to blood and cannot be measured reliably using methods such as ELISA that commonly are applied for serum and urine samples. We also considered that with hormonal contraceptive use, the transcriptome profile would be changed in saliva and that such changes could serve as a potential biomarker of contraceptive exposure.  We speculated that we could use RNA sequencing to identify differentially expressed genes (DEGs) in saliva samples obtained from individuals using hormonal contraception^22–25^.

Accordingly, we conducted a cross-sectional, open-label, pilot study to determine if we could use validated tests to measure hormones or their metabolites in urine specimens obtained from individuals using COCs or DMPA. We also explored the feasibility of using RNA sequencing with saliva samples to detect differentially expressed genes as a marker of hormonal contraceptive use.

## Materials and methods

### Ethical approaches and study population

The study was approved by the Institutional Review Board of the Population Council, the Ethics Committee of Profamilia (study site) in the Dominican Republic, and CONABIOS, the national medical research agency of the Dominican Republic. Each participant provided written informed consent before undergoing any screening procedures. The study was conducted in accordance with the Declaration of Helsinki, the International Conference on Harmonization Good Clinical Practice Guidelines, and the US Code of Federal Regulations pertaining to clinical studies.

We recruited women interested in starting DMPA or COCs for the duration of the study and enrolled two groups (15 per group). Individuals in Group A used a COC containing ethinyl estradiol and LNG (EE: 30 mcg and LNG 150 mcg Microgynon-Bayer, Germany) and those in Group B used DMPA 150 mg (Pfizer Manufacturing Belgium NV, Belgium) administered intramuscularly. Healthy, non-pregnant, non-breastfeeding adults (18–39 years) not currently using DMPA and able to provide written informed consent were eligible. We excluded potential participants if they were enrolled in any other study involving contraceptive products currently or in the 2 months prior to screening, had contraindications to the use of the selected contraceptives, were experiencing vaginal bleeding (not including spotting between periods), tested positive for HIV or hepatitis B infection at screening, or had used DMPA in the past 12 months. A complete list of inclusion/exclusion criteria is presented in Supplementary Table 1S.

### Study procedures, rationale for sampling timepoints, and assessments

All participants had three visits after screening; the timeframe for collecting serum, urine and saliva samples varied by group/contraceptive method (Table 1). Participants in Group A took their first, second and third pills at the study site. They had serum, urine, and saliva collected prior to receiving their first dose and at designated intervals on Days 1 and 3. Participants in Group B had serum, urine, and saliva sampling prior to receiving their first DMPA injection on Day 1, and again on Days 21 and 60 (± 4 days). Site personnel monitored participants for the occurrence of adverse events (AEs) and serious adverse events (SAEs) throughout the study. Data from participants were de-identified in all study documents, samples, analyses, and subsequent calculations.

**Table 1:** Sample collection timepoints from participants using COCs or DMPA.

Method	Visit/day/dose	Time point	Specimen collection		
			Blood	Urine	Saliva
COC	1/1	Before dose 1	X	X	X
		6 h	X	X	X
	2/2	No specimen collected			
	3/3	24 h post dose 2/before dose 3	X	X	X
		6 h	X	X	X
DMPA	1/1	Before injection	X	X	X
	2/21		X	X	X
	3/60		X	X	X

Serum, urine, and saliva sample collection procedures and processing.

Sample collections from participants using COC or Depo. The table shows each type of contraceptive method used and the type of sample: blood, urine, and saliva collected at various time intervals.

We selected time points for collecting urine and serum samples from COC users based on the established pharmacokinetics (PK) of COCs that entail daily peak and trough concentrations of progestin and are the same for each 24-h period during the cycle days that the pill is taken to suppress ovulation^26^. The peak serum concentration of orally administered LNG is generally around one-two h after ingestion, and its metabolites together with small quantities of non-metabolized LNG appear in the urine approximately four to eight hours after ingestion. Therefore, we selected a six-hour time-point to collect both serum and urine following COC ingestion and selected Days 1 and 3 to collect samples (Table 1).

We also used established PK data to select serum and urine collection time points for DMPA users^27^. MPA is released slowly from the DMPA injection site into the blood and levels are maintained to achieve ovulation suppression (efficacy) for at least three months. Small quantities of non-metabolized MPA and its metabolites appear in urine at a steady state beginning on Day 3 post-injection and can be measured reliably throughout the extended period of treatment. The MPA peak concentration generally is found in serum around three weeks after injection and levels are maintained to achieve efficacy for at least three months. Hence, we collected serum from DMPA users on Day 21 and selected Day 60 for the second collection (Table 1).

Regarding saliva, the goal was to identify the impact on the transcriptome profile of saliva in response to LNG and MPA use and concentration in serum. Since saliva is a good indicator of plasma levels of various substances such as hormones and pharmaceuticals and salivary mRNA serves as a chemical signature that a particular gene has been expressed^21^, we assumed that we could use the same time points we selected for the collection of serum and urine to collect saliva (Table 1). In selecting these same time points, we were also influenced by findings from studies that examined other steroidal hormones. With testosterone, for example, investigators identified a significant increase in testosterone (levels) in saliva, blood, and urine when administered transdermally^28^.

*Serum* A phlebotomist collected 10 mL of blood per participant that was processed for serum, immediately frozen, and stored at – 20 °C until shipped on dry ice to the Population Council in New York for analysis.

*Urine* We provided participants with sampling containers to collect 20–30 mL of urine at specified time points. These were kept at – 20 °C until they were shipped.

*Saliva* Site personnel instructed participants to refrain from eating, drinking, smoking, or oral hygiene procedures for at least one hour prior to sampling and to spit saliva into a graduated test tube over the course of 5 minutes to collect approximately 5 mL of saliva. To inhibit RNA degradation, the site staff kept collection tubes on ice prior to collecting saliva. Consistent with supplier instructions (Norgen Biotek, CA, USA), site staff added a 2 mL preservative containing a stabilizing reagent immediately following collection and stored the collection tubes at -80°C in preparation for shipping.

#### Measurement of LNG and MPA levels in serum and urine samples by liquid chromatography with tandem mass spectrometry (LC–MS/MS)

Prior to measuring hormone levels in the study samples, we used our standard protocol consistent with FDA guidelines to validate our LCMS methods for measuring LNG and MPA in serum and urine. Specifically, we spiked six concentration levels of calibration standards (STDs) in the range between 0.1 and 10 ng/mL in human serum and separately prepared three levels of quality control samples (QCs) at 0.3, 1, and 7 ng/mL concentrations. We used LNG-d6 or MPA-d6 (5 ng/ml) as the internal standards (IS) respectively and evaluated and validated the following parameters: specificity, linearity, limit of quantitation, accuracy, precision, matrix effects, recovery, and stability. Our results showed that we met the acceptance criteria of all validation parameters as per the protocol.

For the next step, we measured LNG and MPA levels in both serum and urine samples by LC-MS/MS using LNG-d6 or MPA-d6 as the internal standards respectively. To purify LNG or MPA from serum test samples we used the protein precipitation method. Specifically, we added 100 µl of methanol to 100 µl of the test sample containing the IS (5 ng/ml). We vortexed the samples for 15 mins (centrifuged at 14.5 × 10^3^ rpm for 10 mins) and transferred the supernatants to high-performance liquid chromatography (HPLC) vials for injection. In addition, we processed the LNG or MPA standards and the quality control samples that were spiked with IS using the same protein precipitation method.

To purify LNG or MPA from urine samples, we performed solid-phase extraction using Oasis HLB Plus Light cartridges (Waters Corporation, Milford, MA). We eluted LNG or MPA from the cartridge using 1 ml of methanol and dried each sample under nitrogen gas. We reconstituted the purified LNG/MPA with 150 µl mobile phase A/B (50/50, v/v) and injected 10 µl aliquots into the autoinjector assembly. In addition, we processed LNG or MPA standards, quality control samples spiked with IS and 1 ml blank human urine using the same solid phase extraction method. We used Waters Acquity UPLC BEH C18 (1.7 µm, 2.1 × 50 mm) column to separate LNG or MPA from other components by gradient elution using the mobile phase from 80% A (2 mM ammonium formate + 0.1% formic acetic in 10% methanol) and 20 % B (100% methanol) up to 6 minutes to 90% B with 0.3 ml/min flow rate. We then used Waters TQ-s to monitor the product ion transitions of 313.35 m/z to 245.28 m/z for LNG and 319.38 to 251.32 m/z for LNG-d6 IS. Similarly, we monitored the transitions 387.42 m/z to 327.35 m/z for MPA, and 393.40 m/z to 330.34 m/z for MPA-d6 IS. We used MassLynx software version 4.2 to control all parameters of the LC–MS/MS system.

In analyzing study samples, we included six levels of calibration standards, one double blank, one matrix blank spiked with internal standard, and three levels of quality control samples (n = 2). We placed one set of quality control samples in the front of the sample queue and another set at the end of the sample queue. The calibration standards were injected at the front and reinjected at the end sample queue. The lower limit of quantitation (LLOQ) for serum LNG and MPA was 0.1 ng/ml and for urine LNG and MPA was 25 pg/ml. A 50 µl of the serum sample was used for LNG or MPA in serum assay and a 1 ml human urine sample was used for LNG or MPA in urine assay. Intra- and Inter-assay coefficient of variations was less than 15% based on the results of quality control samples.

#### Measurement of serum and urine LNG levels by enzyme immunoassay (EIA)

We used a DetectX LNG EIA commercial kit (Arbor Assays, MI, USA) to measure LNG and metabolites in serum and urine samples. To validate the assay, we first measured LNG levels in serum samples and compared them to the values obtained by LC–MS/MS. Next, we optimized the measurement of LNG levels in the urine samples that were purified by the same solid phase extraction described for LC–MS/MS measuring procedures. We reconstituted dried residues with an assay buffer and used the immunoassay kit procedure to measure LNG concentrations. We reported urine LNG values as pg/mL; the lower limit of detection was 80 pg/ml.

#### RNA profiling in saliva samples

To extract the total RNA from the saliva of each participant we used TRIzol® lysis buffer, and then purified that RNA using a Nucleospin RNA extraction kit (Takara Bio, CA, USA, Inc). We measured the RNA concentrations using Qubit Fluorometer (Life Technologies, MA, USA) and RNA integrity using the bioanalyzer (Agilent 4200 Tape Station). We conducted RNA library preparations for sequencing reactions. We then labeled RNA and sequenced it using the Total RNA-Seq kit. We used SMART-Seq v4 Ultra Low Input Kit for Sequencing for full-length cDNA synthesis and amplification (Clontech, CA, USA), and Illumina Nextera XT library for sequencing library preparation. Briefly, cDNA was fragmented, and the adaptor was added using Transposase, followed by limited-cycle PCR to enrich and add an index to the cDNA fragments. The final library was assessed with Agilent TapeStation. The samples were sequenced using a 2 × 150 Paired-End (PE) configuration. Using services from GENEWIZ, LLC. (South Plainfield, NJ, USA), we conducted the bioinformatics analysis and applied HiSeq Control Software (HCS) to conduct image analysis and base calling. We converted raw sequence data (.bcl files) generated from Illumina HiSeq into fastq files and de-multiplexed using Illumina's bcl2fastq 2.17 software. One mismatch was allowed for index sequence identification. After demultiplexing, we checked sequence data for overall quality and yield. Then, we trimmed sequence reads to remove possible adapter sequences and nucleotides with poor quality using Trimmomatic v.0.36. We mapped the trimmed reads to the Homo sapiens GRCh38 reference genome available on ENSEMBL using the STAR aligner v.2.5.2b that uses a spliced aligner to detect splice junctions and incorporate them to help align the entire read sequences, resulting in BAM files. We then applied the Subread software package v.1.5.2, which uses feature counts to calculate unique gene hit counts. We only counted unique reads that fell within exon regions, and used the gene hit counts table to analyze downstream differential expression.

### Data analysis

The primary study endpoint was the sensitivity and specificity of LNG or MPA detection in urine. We calculated sensitivity as the total number of urine samples with detectable LNG or MPA by LC–MS/MS divided by the number of serum samples with detectable LNG or MPA per time point. We calculated specificity as the number of urine samples with undetectable LNG or MPA divided by the number of serum samples with undetectable LNG or MPA by time point. We also calculated the sensitivity and specificity of urine LNG measurements by EIA. We calculated 95% confidence intervals based on exact tests around each sensitivity and specificity estimate.

As the exploratory endpoint, we looked for evidence in the saliva of differential expression of specific genes following volunteers’ intake of COCs or injection with MPA. We used DESeq2 to compare gene expression at baseline (pre-dose) with results at post-dose intervals from the saliva samples of both COC and MPA users. By applying the Wald test, we generated p-values and Log2 fold changes. We also identified genes with p-values below the Benjamini–Hochberg adjusted critical values. We used these results to adjust for multiple comparisons and to maintain an overall type 1 error rate below < 0.05 and absolute log2 fold changes > 1 to determine DEGs for each comparison. We then generated volcano plots using the top 500 genes, selected by Log2 fold change and adjusted p-values. We performed a gene ontology analysis on the statistically significant set of genes via the software GeneSCF v.1.1-p2. We used the gene ontology annotation (GOA) human GO list to cluster the set of genes based on their biological processes and determined their statistical significance. We generated a list of genes clustered based on their gene ontologies.

## Results

**Figure 1:**
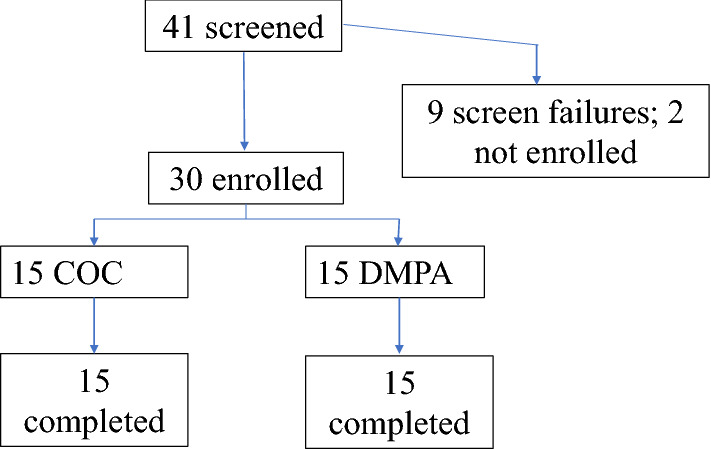
Study Flow Diagram. Consort Diagram showing that 41 potential participants were screened and 30 were enrolled, out of which 15 per group (COC or Depo).

Between August and November 2019, we screened 41 potential participants and enrolled 30, 15 per group (Fig. 1). All participants were Hispanic, of mixed race, parous and had a median age of 29.5 years old (Table 2). All 30 participants completed the study by December 2019 with no SAEs or AEs leading to early discontinuations.

**Table 2:** Demographic characteristics of study participants by group (N = 30).

	Group A. COCs n (%)	Group B. DMPA n (%)	Overall n (%)
Age median (range)	34.3 (26–39)	24.6 (18–39)	29.5 (18–39)
**Race**			
Mixed race (Black and White)	14 (93%)	15 (100%)	29 (97%)
Black	1 (7%)		1 (3%)
Hispanic ethnicity	15 (100%)	15 (100%)	30 (100%)
Parous (had children)	14 (93%)	11 (73%)	26 (87%)
Median number of children	3	1	3
Cohabitating/married	60%	60%	60%
Previous injectable use	80%	60%	70%
Previous COC use	73%	47%	60%

**Figure 2:**
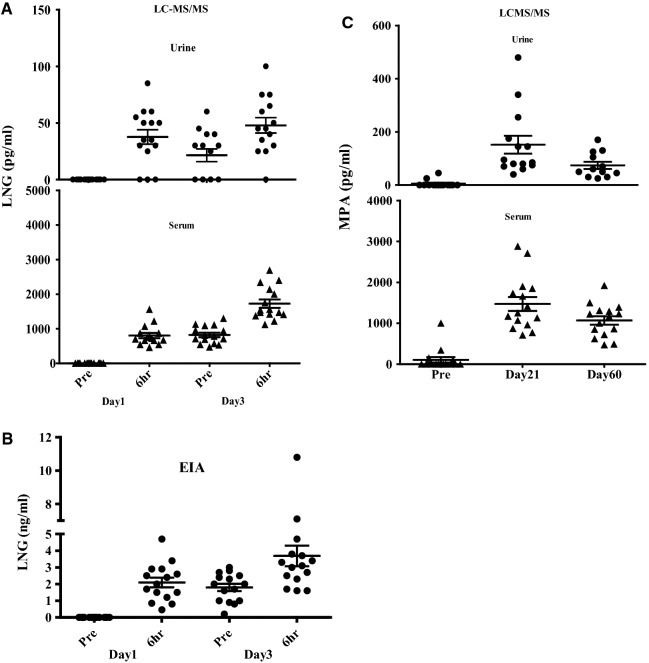
(**A**) Levonorgestrel (LNG) concentrations measured by LCMS/MS method in serum and urine samples were collected on days-1 and 3 of the COC treatment. No individuals showed detectable LNG in serum or urine at baseline (Pre) but all showed an increase at 6 h post ingestion on both days (day-1 and 3). Only eight of 15 participants had detectable levels at all three time points post baseline. Six participants had LNG levels below the level of quantification at the trough timepoint (24 h post dose 2), three of whom also had undetectable levels 6 h after their first dose. (**B**) Levonorgestrel (LNG) concentrations measured by Enzyme Immuno assay (EIA) method in urine samples collected on day-1 and 3 of the COC treatment. No individuals showed detectable LNG in serum or urine at baseline (Pre) and showed detectable levels at all time points post ingestion on both days (day-1 and 3). (**C**) Medroxyprogesterone (MPA) concentrations measured by LC–MS/MS method in serum and urine samples collected on day 0 (Baseline) and 21 and 60 days after depo injection. Four individuals had detectable MPA in serum prior to receiving their injection, two of whom also had detectable MPA in urine. The two women with undetectable urine MPA but detectable serum MPA had levels below that required for contraceptive efficacy (< 0.2 ng/mL). One individual with undetectable serum MPA, had detectable urine MPA, with the level in the urine slightly above the serum threshold level (< 0.2 ng/mL). We detected MPA in serum and urine of all participants at 21- and 60-days post injection.

Sensitivity/Specificity of LNG in urine: In Figs. 2A and B we have presented the quantified serum and urine values for Days 1 and 3, respectively. Table 3 shows LC–MS/MS and EIA sensitivity and specificity results for urine LNG measurements from COC users. With both methods, we observed 100% specificity, with no individuals having detectable LNG in serum or urine at baseline (prior to COC ingestion). Following COC ingestion, serum LNG was detectable for all participants at all collection time points. Sensitivity of urine LNG per LC–MS/MS analysis on Day 1 was 80% six hours post Dose 1 and on Day 3 was 60% 24 hours post Dose 2 and 93% six hours post Dose 3; sensitivity with EIA was 100% at all time points.

**Table 3:** Sensitivity and specificity of detecting LNG in urine samples of COC users (n = 15).

	Day 1		Day 3	
	Pre-dose 1 (baseline)	6 h post-dose 1	24 h post-dose 2/pre-dose 3	6 h post-dose 3
Specificity (LCMS/MS)	100%	–	–	–
Sensitivity (LCMS/MS)	–	80% 95% CI [60–100%]	60% 95% CI [35–85%]	93% 95% CI [81–100%]
Specificity (EIA)	100%			
Sensitivity (EIA)		100%	100%	100%

LC–MS/MS results showed that all participants had at least one time-point when LNG was detectable in urine. Only eight of 15 participants had detectable levels at all three time points post baseline (Fig. 2A). Six participants had LNG levels below the level of quantification at the trough timepoint (24 h post Dose 2), three of whom also had undetectable levels six hours after their first dose (Fig. 2A). All but one participant had LNG detected per LC–MS/MS in urine six hours after the post dose three. Notably, that individual had urine LNG detected after the first and second timepoints. Actual LNG levels for all participants using LCMS/MS and EIA are presented in Table 2S.

Table 4 shows LC–MS/MS detection of MPA in urine and serum samples for MPA users. Of the 11 participants with undetectable MPA in serum at baseline, 10 had samples with undetectable MPA in urine, corresponding to 91% specificity pre-dose in urine. Four individuals had detectable MPA in serum prior to receiving their injection (Fig. 2C), two of whom also had detectable MPA in urine. Both individuals with MPA detected in both serum and urine denied any prior DMPA injections. The two participants with undetectable urine MPA but detectable serum MPA had levels below that required for contraceptive efficacy (< 0.2 ng/mL). They reported having received DMPA injections more than 12 months prior to study start (15 and 17 months, respectively). The individual with undetectable serum MPA had detectable urine MPA, with the level in the urine slightly above the serum threshold level (< 0.2 ng/mL). We detected MPA in serum and urine of all participants at 21- and 60-days post injection (100% sensitivity). In Table 3S we have illustrated the actual MPA levels for all participants using LC–MS/MS.

**Table 4:** Sensitivity and specificity of detecting MPA in urine samples of DMPA users (n = 15).

	Baseline	Day 21	Day 60
Specificity (LCMS/MS)	91% CI [59–100%]*	–	–
Sensitivity (LCMS/MS)	–	100%	100%

*n = 11 samples with undetectable MPA at baseline.

MPA Detection in urine and serum samples of Depo-Provera users (n = 15) at Baseline. We observed 91% specificity pre-dose in urine. 10/11 women (91%) had undetectable MPA at baseline. The serum level for 2 out of 4 women was < 0.2 ng/ml.

**Figure 3:**
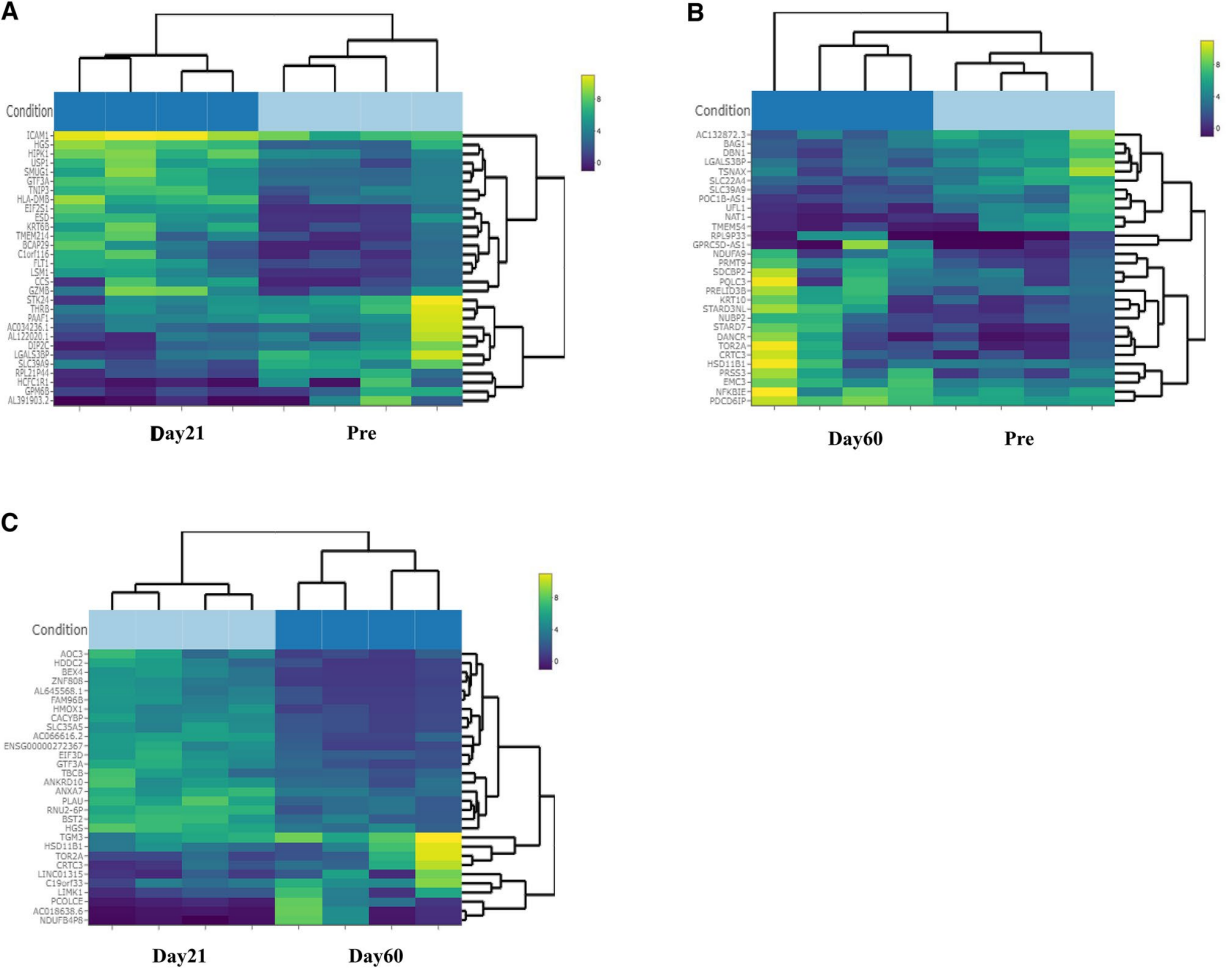
(**A**–**C**) A bi-clustering heatmap showing the visual expression profile of the top differentially expressed genes sorted by their adjusted p-value by plotting their log2 transformed expression values in samples (generated by using the Wald test). (**A**) shows the pre- versus D21 comparison where we observed 10 differentially expressed genes, out of which nine were upregulated and one was downregulated. (**B**) shows pre-versus D60 comparison, we saw five differentially expressed genes; one was downregulated and four were upregulated. (**C**) shows the last comparison between D21 versus D60, there were a total of 50 differentially expressed genes out of which 32 were downregulated and 18 were upregulated.

**Figure 4:**
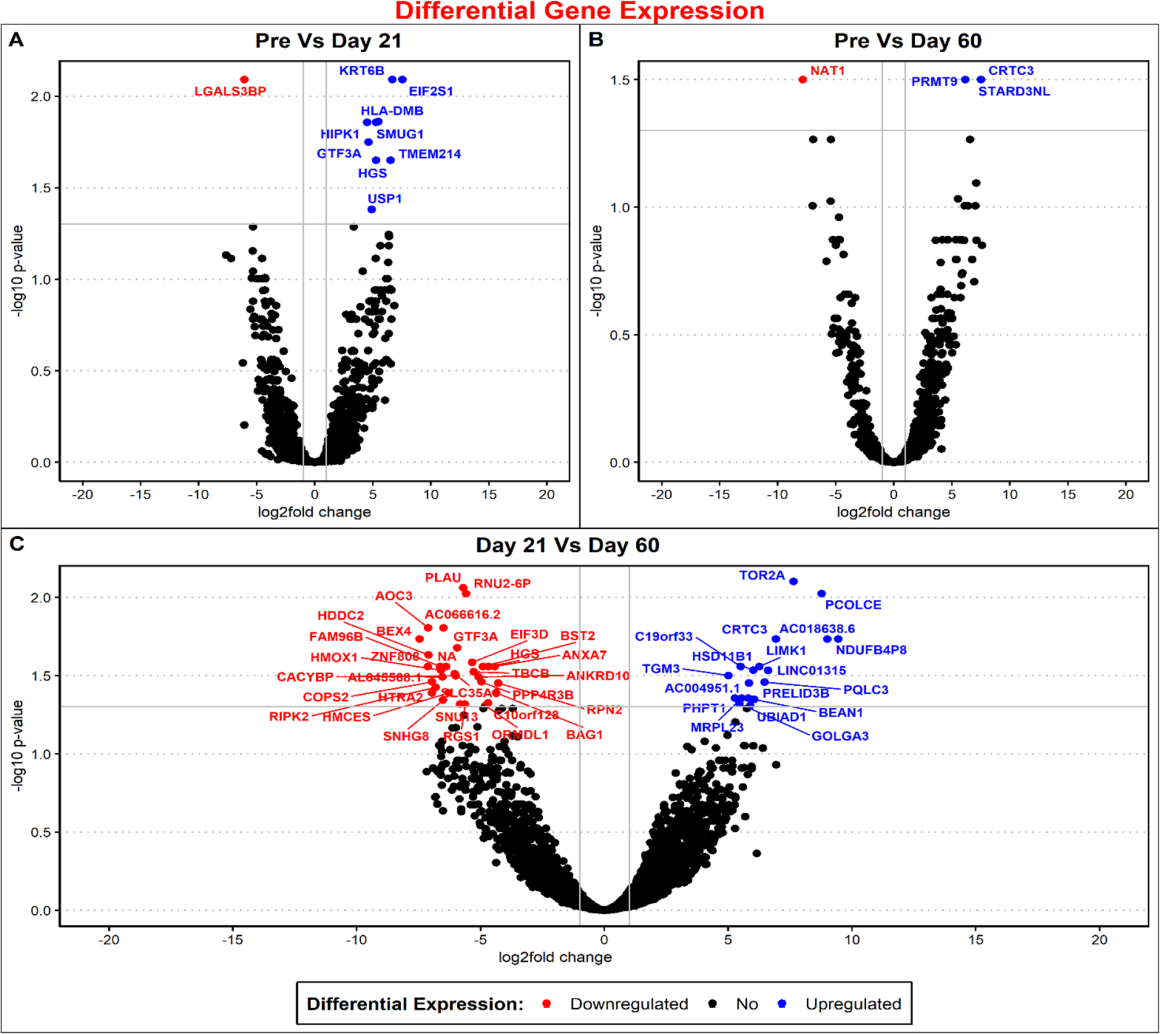
(**A**–**C**) Volcano plots to visualize the expression profile of the top differentially expressed genes sorted by their adjusted p-value by plotting their log2 transformed expression values. Downregulated DEGs are noted in red and upregulated DEGs are noted in blue.

Our analysis of RNA sequencing of saliva samples from the participants who used DMPA revealed varying numbers of DEGs at the three timepoints (Table 5). The information in the bi-clustering heatmaps (Fig. 3A–C), illustrate the visual profile of the DEGs from samples obtained at Days 21 (D21) and 60 (D60) compared to baseline (pre) samples and to each other, sorted by their adjusted p-values, and plotted with their log2 transformed expression values. For the pre-versus D21 comparison (3a) we observed 10 differentially expressed genes; nine of these genes were upregulated and one was downregulated. For pre- versus D60 (3b) we saw five DEGs; one was downregulated and four were upregulated. For the last comparison between D21 versus D60 (3c) there were a total of 50 differentially expressed genes out of which 32 were downregulated and 18 were upregulated. The volcano plots of gene expression in saliva of DMPA users according to the fold change and adjusted p values depict DEGs at different time intervals (Fig. 4A–C). We have listed the differentially expressed genes identified by their adjusted p values plus their standard errors and confidence intervals (CIs) in Table 4S. We did not detect any differentially expressed genes in saliva samples of COC users compared to baseline.

**Table 5:** Total number of differentially expressed genes in saliva sample of DMPA users.

Comparison	Upregulated genes	Downregulated genes	Total significantly differentially expressed genes
PRE-vs-day 21	9	1	10
PRE-vs-day 60	3	1	4
Day 21-vs-day 60	18	32	50

Total number of differentially expressed genes in DMPA users at different time points versus baseline.

## Discussion

This study represents a novel endeavor within the field of contraception to confirm individual use of hormonal methods by measuring biomarkers in urine and saliva samples. We were able to demonstrate that we can measure native hormones (LNG or MPA) at low concentrations in urine using LC–MS/MS) in samples obtained from participants who used COCs or DMPA. The sensitivity for LNG was 93% [68–100%] at 6 h post-dose on Day 3 and sensitivity for MPA was 100% on Days 21 and 60; specificity was 91% [59–100%]. Our results provide strong support for proceeding with additional research to confirm our findings regarding using urine sampling to identify a biomarker of hormonal contraceptive use. We propose synthesizing the reference metabolites present in urine for both LNG and MPA and measuring urine metabolite concentrations by LC–MS/MS. Metabolite(s) concentrations are expected to be much higher than the intact hormones^29–31^, thus we are confident that measuring specific urine metabolites will enhance sensitivity and specificity and foster further development of using urine sampling as a biomarker of hormonal contraceptive use.

To our knowledge, our results are the first that demonstrate the ability to measure LNG concentrations in urine samples using the DetectX kit. As compared to our results using LC–MS/MS technology, urine LNG concentrations measured by EIA were much higher. This result was not surprising as the EIA LNG method is based on polyclonal antibodies that bind to both LNG and its metabolites. We need to confirm if this EIA outcome was due to the cross-reaction of LNG metabolites present in urine with the polyclonal anti-LNG antibodies used in the DetectX kits and propose synthesizing the specific reference metabolite(s) to further validate the LNG EIA kit for correct measurement of LNG and metabolites in urine. Similarly, the reference metabolites will help in increasing the sensitivity/specificity of measuring metabolites in urine by EIA, which may be a more cost effective and accessible method for use in low resource settings compared to LC–MS/MS technology.

Our exploration of saliva samples provides preliminary evidence that saliva could be used to identify a biomarker of hormonal contraceptive use. Based on the recent identification of differential gene expression in saliva samples obtained from cancer patients, which has paved the way for exploring saliva as a biomarker for various conditions^32–34^, we hypothesized that hormonal contraceptive use could lead to up-or down-regulation of some selective genes in individuals using DMPA or a COC. As illustrated in the heatmaps, we found numerous differentially expressed genes in the group who used DMPA on Day 21 and Day 60 compared to baseline, the majority of which have been described previously in association with various disease entities or conditions. Interestingly, in our study, the changes we noted appear to align with adverse effects that some individuals report when using DMPA. Both weight gain^35^ and alopecia^36^, for example, are reported among some DMPA users, and our analysis of differential gene expression showed that the CRTC (CREB-regulated transcription coactivator 3) gene, which plays a role in obesity^35^ and PRMT9 (Protein Arginine Methyltransferase 9) gene that has been associated with mandibulofacial dysostosis with alopecia were both up-regulated. Additionally, we identified that N-acetyltransferase1, which functions in folate catabolism^37,38^, was downregulated. This finding is consistent with a Centers for Disease Control report noting that DMPA users are likely to be ranked in the lowest quartile (≤ 6.2 ng/mL) for serum folate concentrations^39^. We also identified that lgals3bp galectin-3-binding protein, which is regarded as an important clinical tumor biomarker^40–42^, was downregulated in DMPA users. Interestingly, DMPA use has been reported to induce a dramatic decrease in endometrial epithelial proliferation and is even suggested as a chemo-preventive agent in individuals with Lynch syndrome^43^. Further confirmatory studies are required to demonstrate if any of these differentially expressed genes identified for these other conditions could also serve as a biomarker in the saliva of DMPA users.

In contrast, we did not detect any differential gene expression in saliva samples of COC users collected at 24- and 72-h post-ingestion. The negative result could have been due to the short time points and sequences we followed for the saliva sampling of COC users. Likely the changes in mRNA levels in COC users were comparatively small after only 24 and 72 h of use. The ability to accurately assess the presence of hormones in saliva depends on the biochemical nature of the hormones and the mechanism by which the hormones, e.g., LNG, enter the oral cavity^44,45^. Therefore, the undetectable gene changes in COC users after 24–72 h of use may have been related to the specific transport mechanism of LNG into saliva, which needs to be explored further.

In addition, in this study, we only performed total RNA sequencing that primarily detects coding RNA. Results from recent studies have demonstrated that long non-coding RNA and micro-RNA can interact through various mechanisms to regulate mRNA^46^. Future research efforts wherein we apply longer periods of COC use (and saliva collection times) as well as microRNA sequencing that query thousands of small noncoding RNA sequences with unprecedented sensitivity may enable us to identify differentially expressed genes in COC users.

This study had several strengths as well as limitations. Our results demonstrating that urine sampling can be used to identify a biomarker of hormonal contraceptive use have set the stage for further development, including a path towards identification of diagnostic methods using urine sampling that be applied easily and widely. The fact that we did not utilize derivatization technology to measure hormone levels was a study limitation. With this method, it is likely that we could have increased the detection of lower levels of hormones in urine samples and may have improved the sensitivity of our results for detecting both COC and DMPA use. Another limitation was that we did not measure volunteers’ baseline blood levels of LNG/MPA prior to initiating treatment with COCs or DMPA. These data points could have improved our specificity results obtained from the urine samples.

Regarding our results with saliva, our positive results in DMPA users suggest that we may be able to use this easily obtained body fluid to identify biomarker(s) of hormonal contraceptive use. Using RNA sequencing technology only for this phase of the study may have prevented us from showing any differential expression in the saliva of COC users and was a study weakness. Additionally it is possible that changes in salivary gene expression are cumulative, and therefore the short time frame we used to explore such changes represented an additional weakness. We will need to conduct further research to assess the effects of longer exposure to COC use on differential gene expression, and apply micro RNA sequencing to obtain additional information to determine if further exploration is warranted for using saliva sampling to detect biomarkers of COC use.

Overall, the possibilities raised by our results suggest that we may be able to use urine or saliva to develop novel biomarkers indicative of hormonal contraceptive use that could be applied in public health research and also have implications for clinical research and clinical care.

## Supplementary Information

Supplementary Tables.

## References

[1] Sustainable Development Goals and Family Planning 2020: International Planned Parenthood Federation (IPPF). https://www.ippf.org/sites/default/files/201611/SDG%20and%20FP2020.pdf. Accessed 20 Feb 2018.

[2] Ahmed S (2019). Trends in contraceptive prevalence rates in sub-Saharan Africa since the 2012 London Summit on Family Planning: Results from repeated cross-sectional surveys. Lancet Glob. Health..

[3] Beatty A (2015). The determinants of recent trends in fertility in Sub-Saharan Africa. National Acad. Sci. Eng. Med..

[4] Brown MT (2016). Medication adherence: Truth and consequences. Am. J. Med. Sci..

[5] Leahy ME, Bernice K, John R (2015). Tracking changes in states of contraceptive use over time in Sub-Saharan Africa through cohort and period analyses. J. Biosoc. Sci..

[6] Lapane KL, Dubé CE, Schneider KL, Quilliam BJ (2007). Misperceptions of patients vs providers regarding medication-related communication issues. Am J Manag Care..

[7] Dehlendorf C, Grumbach K, Schmittdiel JA, Steinauer J (2017). Shared decision making in contraceptive counseling. Contraception.

[8] Center for Drug Evaluation and Research. Guideline for submitting supporting documentation in drug applications. *U.S. Food and Drug Administration*. https://www.fda.gov/regulatory-information/search-fda-guidance-documents/guideline-submitting-supporting-documentation-drug-applications-manufacture-drug-products.

[9] Achilles SL (2018). Misreporting of contraceptive hormone use in clinical research participants. Contraception.

[10] Tsui AO (2021). Is client reporting on contraceptive use always accurate? Measuring consistency and change with a multicountry study. Stud. Fam. Plann..

[11] Houston L, Yu P, Martin A (2021). Clinical researchers’ lived experiences with data quality monitoring in clinical trials: A qualitative study. BMC Med. Res. Methodol..

[12] Archer D (2019). Efficacy of the 1-year (13-cycle) segesterone acetate and ethinylestradiol contraceptive vaginal system: results of two multicentre, open-label, single-arm, phase 3 trials. BMC Med. Res. Methodol..

[13] Hall KS, O'Connell White K, Reame N, Westhoff C (2010). Studying the use of oral contraception: A review of measurement approaches. JWH..

[14] Pyra M (2018). Concordance of self-reported hormonal contraceptive use and presence of exogenous hormones in serum among African women. Contraception.

[15] Nwaohiri AN (2018). Discordance between self-reported contraceptive use and detection of exogenous hormones among malawian women enrolling in a randomized clinical trial. Contraception.

[16] Castaño PM (2012). Effect of daily text messages on oral contraceptive continuation. Obstet. Gynecol..

[17] Langhaug LF, Sherr L, Cowan FM (2010). How to improve the validity of sexual behavior reporting: Systematic review of questionnaire delivery modes in developing countries. Tropical Med. Int. Health.

[18] Tourangeau R, Rips LJ, Rasinski K (2000). The Psychology of Survey Response.

[19] Stanczyk FZ, Clarke NJ (2010). Advantages and challenges of mass spectrometry assays for steroid hormones. J. Steroid Biochem. Mol. Biol..

[20] Newman M, Pratt SM, Curran DA (2019). Evaluating urinary estrogen and progesterone metabolites using dried filter paper samples and gas chromatography with tandem mass spectrometry (GC–MS/MS). BMC Chem..

[21] Tiwari M (2011). Science behind human saliva. J. Nat. Sci. Biol. Med..

[22] Vining RF, McGinley RA, Symons RG (1983). Hormones in saliva: Mode of entry and consequent implications for clinical interpretation. Clin. Chem..

[23] Liu J (2018). Quantification of 10 steroid hormones in human saliva from Chinese adult volunteers. J. Int. Med. Res..

[24] Kaczor-Urbanowicz KE (2016). Saliva diagnostics: Current views and directions. Exp. Biol. Med..

[25] Spielmann N, Wong DT (2010). Saliva: Diagnostics and therapeutic perspectives. Oral Dis..

[26] Hofmann BM (2020). Comparative pharmacokinetic analysis of levonorgestrel-releasing intrauterine systems and levonorgestrel-containing contraceptives with oral or subdermal administration route. Eur. J. Contracept. Reprod. Health Care.

[27] Ortiz A, Hiroi M, Stanczyk FZ, Goebelsmann U, Mishell DR (1977). Serum medroxyprogesterone acetate (MPA) concentrations and ovarian function following intramuscular injection of Depo-MPA. J. Clin. Endocrinol. Metab..

[28] Schönfelder M (2016). Potential detection of low-dose transdermal testosterone administration in blood, urine, and saliva. Drug Test. Anal..

[29] Marylouise H, Roberta H (1962). Identification of a 6,21-dihydroxlyated metabolite of medroxyprogesterone acetate in human urine. J. Clin. Endocrinol. Metab..

[30] Alton KB, Hetyei NS, Shaw C, Patrick JE (1984). Biotransformation of norgestimate in women. Contraception.

[31] Stanczyk FZ, Roy S (1990). Metabolism of levonorgestrel, norethindrone, and structurally related contraceptive steroids. Contraception.

[32] Yoshizawa JM (2013). Salivary biomarkers: Toward future clinical and diagnostic utilities. Clin. Microbiol. Rev..

[33] Goswami Y, Mishra R, Agrawal AP, Agrawal LA (2015). Salivary biomarkers: A review of powerful diagnostic tool. J. Dent. Med. Sci..

[34] Wang X, Kaczor-Urbanowicz KE, Wong DT (2016). Salivary biomarkers in cancer detection. Med. Oncol..

[35] Moreno CL (2016). Role of hypothalamic creb-binding protein in obesity and molecular reprogramming of metabolic substrates. PLoS ONE.

[36] PRMT9 Gene Card. GeneCards. https://www.genecards.org/cgi-bin/carddisp.pl?gene=PRMT9.

[37] Sim E, Abuhammad A, Ryan A (2014). Arylamine N-acetyltransferases: From drug metabolism and pharmacogenetics to drug discovery. Br. J. Pharmacol..

[38] Laurieri N (2014). From arylamine N-acetyltransferase to folate-dependent acetyl CoA hydrolase: Impact of folic acid on the activity of (HUMAN)NAT1 and its homologue (MOUSE)NAT2. PLoS ONE.

[39] CDC (2000). Serum folate levels among women attending family planning clinics: Georgia. MMWR.

[40] Piccolo E (2012). LGALS3BP, lectin galactoside-binding soluble 3 binding protein, induces vascular endothelial growth factor in human breast cancer cells and promotes angiogenesis. J. Mol. Med..

[41] Lodermeyer V (2018). The antiviral activity of the cellular glycoprotein LGALS3BP/90K is species specific. J. Virol..

[42] Stampolidis P, Ullrich A, Iacobelli S (2015). LGALS3BP, lectin galactoside-binding soluble 3 binding protein, promotes oncogenic cellular events impeded by antibody intervention. Oncogene.

[43] Lu KH (2013). Prospective, multi-center randomized intermediate biomarker study of oral contraceptive vs. depo-provera for prevention of endometrial cancer in women with lynch syndrome. Cancer Prev. Res..

[44] Greabu M (2009). Saliva: A diagnostic window to the body, both in health and in disease. J. Med. Life..

[45] Miočević O (2017). Quantitative lateral flow assays for salivary biomarker assessment: A review. Front. Public Health.

[46] López-Urrutia E (2019). Crosstalk between long non-coding RNAs, micro-RNAs and mRNAs: Deciphering molecular mechanisms of master regulators in cancer. Front. Oncol..

