# Supplementary material for: New approaches for developing biomarkers of hormonal contraceptive use
Source: Sci Rep. 2023 Jan 5;13:245. doi: 10.1038/s41598-022-24215-4 (PMC9816169; doi:10.1038/s41598-022-24215-4)
Supplement: Supplementary file 1 — Supplementary Tables. [file 41598_2022_24215_MOESM1_ESM.pdf]

Table 1s: Listing of Inclusion and Exclusion Criteria:

Inclusion Criteria:	Exclusion Criteria:
• Between 18 and 39 years of age (at screening), inclusive.<br>• Not pregnant based on urine pregnancy tests at screening and enrollment.<br>• Willing to use COCs (Group A); or willing to initiate Depo-Provera use (Group B)<br>• Women who have had a tubal ligation may be enrolled in Group A<br>• Women who have used Depo Provera more than 12 months prior to screening may be enrolled in Group B.<br>• Healthy based on medical history, physical exam and pelvic exam.<br>• Willing and able to provide written informed consent.<br>• Able to follow study procedures.	• Currently pregnant or breastfeeding, or within 3 months post-pregnancy outcome.<br>• Participation in any other clinical research trial involving investigational or marketed contraceptive products currently, or in the 2 months prior to screening.<br>• Undiagnosed vaginal bleeding (not including spotting between periods).<br>• History of hysterectomy or menopause.<br>• HIV or hepatitis B infection identified at screening.<br>• Use of any other type of of hormonal contraceptive in the 3 months before screening, except for the methods being used in this investigation.<br>• Use of Depo-Provera in the past 12 months<br>• History of gynecological surgery or procedure in the 2 months prior to screening.<br>• Breast cancer or other estrogen- or progestin-sensitive cancer, now or in the past.<br>• Women with HSIL on Pap smear taken in the past 2 years.<br>• Known or history of thromboembolic disorders or cerebral vascular disorder.<br>• History of liver dysfunction or diagnosis.<br>• Known hypersensitivity to Depo-Provera or hormonal contraceptives containing ethinyl estradiol and/or a progestin.<br>• Known current drug abuse, including illicit drugs, or alcohol abuse.<br>• Unable to comply with study requirements, including but not limited to, attending all study visits, and use of allowable effective contraceptives.<br>• Any other condition(s) that, in the opinion of the Investigator, might interfere with adherence to trial requirements or evaluation of the trial objectives.

Table 2s – Actual LNG levels for all the participants using LC-MS/MS and EIA

LNG (pg/ml) - LC-MS/MS																	
Urine	Day 1	Pre	0	0	0	0	0	0	0	0	0	0	0	0	0	0	0
		6Hr	25	0	35	55	30	35	0	50	50	0	30	85	60	50	60
	Day 3	Pre	25	0	0	40	0	30	0	0	60	0	30	30	40	45	50
		6Hr	0	30	75	40	35	50	25	25	75	45	65	100	45	60	60
Serum	Day 1	Pre	0	0	0	0	0	0	0	0	0	0	0	0	0	0	0
		6Hr	660	540	657	866	749	871	458	703	1070	721	1560	539	671	1220	770
	Day 3	Pre	950	470	780	928	565	1110	690	710	543	810	1300	534	710	1130	1090
		6Hr	1750	1120	1470	2140	1470	2400	1370	1540	1560	1420	2690	1220	1410	2340	2000

LLOQ (lower limit of quantification) < 25 pg/ml

LNG (ng/ml) - EIA																	
Urine	Day 1	Pre	0	0	0	0	0	0	0	0	0	0	0	0	0	0	0
		6Hr	1.70	2.90	4.70	2.90	2.50	0.46	0.85	2.60	0.80	1.20	2.40	3.40	1.50	1.50	2.00
	Day 3	Pre	2.00	2.00	1.60	2.80	1.00	0.90	1.00	0.20	1.70	2.50	0.80	2.70	2.40	3.00	2.30
		6Hr	3.10	7.10	10.80	3.40	2.50	1.60	1.60	1.70	4.70	2.70	2.30	3.80	3.30	3.70	3.00

LLOQ (lower limit of quantification) < 0.08 ng/ml

Table 3s – Actual MPA levels for all the participants using LC-MS/MS

MPA (pg/ml) - LC-MS/MS																
**Urine**	**Pre**	**0**	**0**	**0**	**30**	**0**	**0**	**45**	**0**	**0**	**0**	**0**	**0**	**0**	**0**	**0**
	**Day21**	**340**	**480**	**70**	**60**	**145**	**95**	**85**	**75**	**80**	**40**	**175**	**255**	**80**	**145**	**200**
	**Day60**	**75**	**30**	**105**	**50**	**45**	**25**	**50**	**30**	**60**	**70**	**170**	**125**	**130**	**75**	**135**
**Serum**	**Pre**	**0**	**0**	**100**	**150**	**0**	**0**	**0**	**0**	**0**	**340**	**0**	**0**	**1000**	**0**	**0**
	**Day21**	**710**	**1660**	**1170**	**1250**	**1130**	**1070**	**1410**	**770**	**960**	**870**	**2880**	**1730**	**1900**	**2710**	**1850**
	**Day60**	**490**	**1000**	**860**	**1170**	**470**	**1500**	**620**	**720**	**1310**	**1230**	**1290**	**1920**	**1220**	**1390**	**850**

LLOQ (lower limit of quantification) < 25 pg/ml

Table 4s: The top differentially expressed genes sorted by their adjusted p-value

PRE -vs- Day21						PRE-vs-Day60						Day21-vs-Day60					
**Gene Name**	**log2Fold Change**	**padj**	**SE**	**CI**		**Gene Name**	**log2Fold Change**	**padj**	**SE**	**CI**		**Gene Name**	**log2Fold Change**	**padj**	**SE**	**CI**	
**LGALS3BP**	-6.06	0.01	1.2948	-3.522	-8.597	**STARD3NL**	7.52	0.03	1.75318	10.956	4.083	**TOR2A**	7.64	0.01	1.574163	10.725	4.554
**EIF2S1**	7.54	0.01	1.5800	10.636	4.443	**CRTC3**	7.5	0.03	1.793009	11.014	3.985	**PLAU**	-5.69	0.01	1.211879	-3.3147	-8.065
**KRT6B**	6.68	0.01	1.4447	9.511	3.848	**PRMT9**	6.15	0.03	1.488312	9.0670	3.232	**PCOLCE**	8.77	0.01	1.922673	12.538	5.001
**HLA-DMB**	5.49	0.01	1.2342	7.909	3.071	**NAT1**	-7.84	0.03	1.87983	-4.155	-11.524	**RNU2-6P**	-5.58	0.01	1.230827	-3.1675	-7.992
**SMUG1**	5.28	0.01	1.2071	7.645	2.914							**AOC3**	-7.11	0.02	1.637286	-3.900	-10.319
**HIPK1**	4.51	0.01	1.0339	6.536	2.483							**AC066616.2**	-6.49	0.02	1.483932	-3.581	-9.398
**GTF3A**	4.62	0.02	1.0823	6.741	2.498							**BEX4**	-7.46	0.02	1.750613	-4.0287	-10.891
**TMEM214**	6.52	0.02	1.5659	9.589	3.450							**CRTC3**	6.93	0.02	1.653145	10.1701	3.689
**HGS**	5.29	0.02	1.2665	7.772	2.807							**NDUFB4P8**	9.45	0.02	2.254528	13.868	5.031
**USP1**	4.9	0.04	1.2279	7.306	2.493							**AC018638.6**	9	0.02	2.132718	13.180	4.819
